# Selective detection of viable seed-borne *Acidovorax citrulli* by real-time PCR with propidium monoazide

**DOI:** 10.1038/srep35457

**Published:** 2016-10-14

**Authors:** Qian Tian, Jian-jun Feng, Jie Hu, Wen-jun Zhao

**Affiliations:** 1 Chinese Academy of Inspection and Quarantine, Beijing 100176, China; 2 Shenzhen Entry-Exit Inspection and Quarantine Bureau, Shenzhen 518045, China; 3 Shenzhen Academy of Inspection and Quarantine, Shenzhen 518010, China; 4 Shaanxi University of Technology, Shaanxi 723001, China

## Abstract

In recent years, use of the DNA-intercalating dye propidium monoazide (PMA) in real-time PCR has been reported as a novel method to detect viable bacteria in different types of samples, such as food, environmental, and microbiological samples. In this study, viable cells of *Acidovorax citrulli*, the causal agent of bacterial seedling blight and fruit blotch, were selectively detected and differentiated from dead cells by real-time fluorescent polymerase chain reaction amplification after the bacterial solution was treated with the DNA-binding dye PMA. The primers and TaqMan probe were based on the *A. citrulli* genome (Aave_1909, Gene ID: 4669443) and were highly specific for *A. citrulli*. The detection threshold of this assay was 10^3^ colony-forming units per mL (CFU/mL) in pure cell suspensions containing viable and dead cells and infected watermelon seeds. Application of this assay enables the selective detection of viable cells of *A. citrulli* and facilitates monitoring of the pathogen in watermelon and melon seeds.

*Acidovorax citrulli*, the causal agent of bacterial seedling blight and fruit blotch (BFB), has emerged as a devastating seed-borne pathogen of watermelon and other cucurbits worldwide, which can cause significant economic losses^1^,^2^,^3^,^4^,^5^. Owing to its economic importance, *A. citrulli* is also regarded as an official import plant quarantine bacterium in the USA, China, and Europe. It is well known that the disease is seed-transmitted and that symptomatic fruit generally contain infected seeds^6^; therefore, the use of pathogen-free seeds and transplants has become an important part of the control strategy. As a result, there is an urgent need for the development of a rapid and accurate method to selectively detect viable, seed-borne *A. citrulli* from seeds. Several methods of *A. citrulli* detection have previously been reviewed including isolation on semi-selective media, seriology, and polymerase chain reaction (PCR)-based methods such as classical PCR, real-time PCR, BIO-PCR, and immunomagnetic separation-PCR^7^,^8^,^9^,^10^,^11^. Compared to traditional cultivation-based practices, PCR-based methods are rapid and sensitive; however, they are limited by their inability to differentiate between DNA from viable organisms and that from dead microorganisms.

In recent years, the DNA-intercalating agent propidium monoazide (PMA) has been used in PCR to overcome problems related to PCR-based methods. PMA is a derivative of propidium iodide that can selectively penetrate into dead cells with compromised membranes and covalently bind DNA upon exposure to an intense light source. In this new approach, samples are treated with PMA prior to DNA extraction. Thus, PMA can specifically inhibit the PCR amplification of DNA derived from dead cells and enable the differentiation of live and dead cells in a bacterial population^12^. Correspondingly, its use in combination with real-time PCR has been effectively applied to detect viable cells in many different microbiological species^13^,^14^,^15^,^16^,^17^,^18^,^19^.

Our objective was to implement the PMA/real-time fluorescent PCR technique for the selective detection of viable, seed-borne *A. citrulli*. For this purpose, we designed new *A. citrulli* species-specific primers and TaqMan probe, and evaluated a novel real-time fluorescent PCR method for the specific and sensitive detection of *A. citrulli*.

## Results

### Specificity and sensitivity of real time-PCR

**Figure 1:**
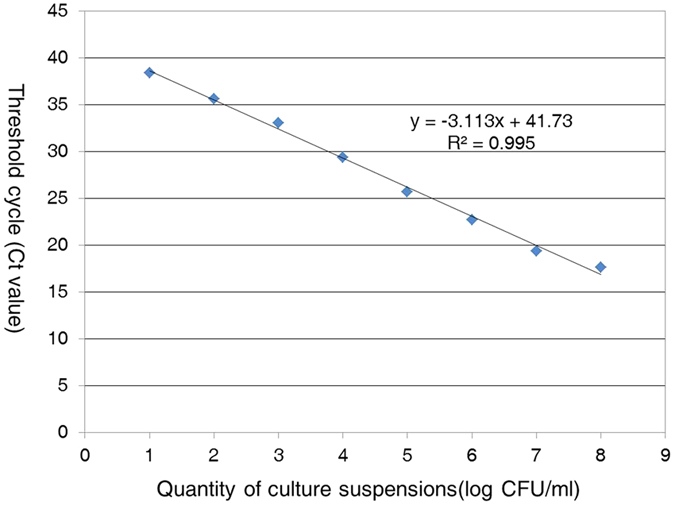
Sensitivity of *A. citrulli* detection using the TaqMan real-time PCR assay. DNA was prepared as serial 10-fold dilutions of *A. citrulli* strain tw24 cell cultures (8 × 10^8^ to 8 × 10 CFU/mL) and used as a template to test the sensitivity of the real-time PCR assay. The standard curve plot, slope, Y-intercept, and R2 are shown.

Specificity analyses for the real-time PCR assay showed positive results for all 116 strains of *A. citrulli*, whereas no reaction was observed for the other 23 bacteria tested including the other closely related *Acidovorax* species *A. avenae*, *A. konjaci*, and *A. cattleyae*. The results of the real-time fluorescent PCR assay are presented in Supplementary Table S1. The relationship between the concentration of *A. citrulli* strain tw24, from 10^8^ to 10 CFU/mL, and the Ct value is shown in Fig. 1. Plotting of the log-concentration of the bacteria versus the cycle number yielded a straight-line regression, with a correlation coefficient (R^2^) of 0.995. The detection limit of the real-time PCR assay with the *A. citrulli* primers/probe set was approximately 8 × 10^3^ CFU/mL in 10-fold serial dilutions of the pure bacterial cell suspension.

### Effect of PMA treatment on real-time PCR amplification of DNA from viable and dead cells from pure bacterial cultures

**Figure 2:**
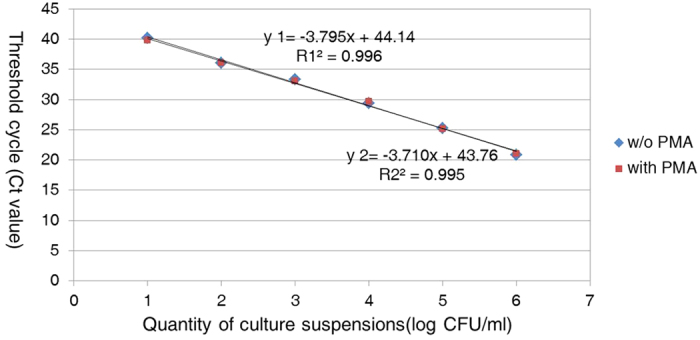
Amplification of DNA from the PMA-treated viable cells by the TaqMan real-time PCR assay. Standard curve plot of real-time PCR generated using 10-fold serial dilutions of PMA-treated and non-treated *A. citrulli* strain tw24 cultures (8 × 10^6^ to 8 × 10 CFU/mL).

To determine whether PMA treatment affected the amplification of viable cell DNA, serial 10-fold dilutions of PMA-treated and non-treated viable *A. citrulli* at concentrations ranging from 8 × 10^6^ to 8 × 10 CFU/mL were compared. Both curves from the PMA-treated and non-treated viable cells were linear and overlapping (Fig. 2), illustrating that PMA treatment had virtually no effect on DNA amplification for viable cells.

**Figure 3:**
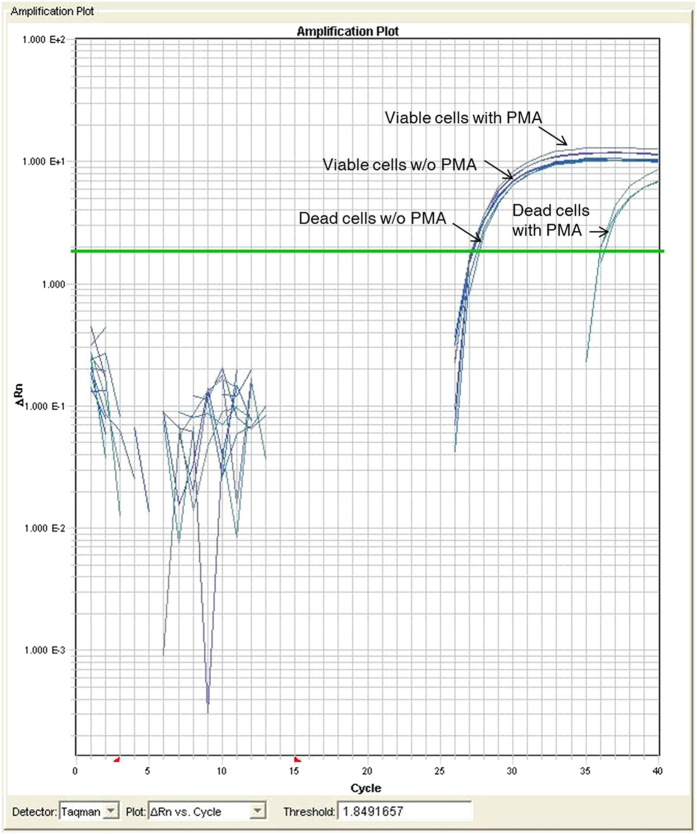
Real-time PCR amplification plots for pure bacterial cultures (10^6 ^CFU/mL). Amplification plot comparison of PMA-treated viable and dead cells with non-treated viable and dead cells using real-time PCR.

Additionally, we compared PMA-treated and non-treated viable and dead cells (approximately 8 × 10^6^ CFU/mL) simultaneously to evaluate the efficacy of PMA treatment to selectively detect viable cells in pure cultures. Similar Ct values were observed for the PMA-treated and non-treated viable cells and for non-treated dead cells. In contrast, there was a 10-Ct difference between the PMA-treated dead cells and the other samples (Fig. 3). This result indicates that the PMA/real-time PCR assay effectively detected both live and dead cells and that PMA specifically inhibited DNA amplification from dead cells.

### Evaluation of PMA/real-time PCR for the detection of viable cells from mixtures of viable and dead cells

**Figure 4:**
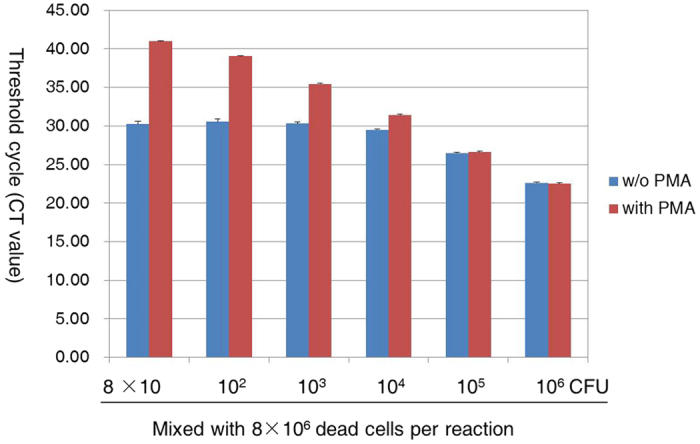
Ct values for viable and dead cell mixtures. Various mixtures of viable cells (8 × 10^6^ to 8 × 10 CFU/mL) and 8 × 10^6 ^CFU/mL dead cells were generated and treated with PMA or left untreated prior to DNA preparation. Each bar represents the average triplicate Ct value.

Various mixtures of viable cells (8 × 10^6^ to 8 × 10 CFU/mL) and 8 × 10^6 ^CFU/mL dead cells were used to evaluate the efficacy of the PMA/real-time PCR assay to selectively detect viable cells in the presence of dead cells. Real-time PCR showed that PMA-treated mixtures displayed a descending trend in Ct that was reciprocal to the number of viable cells, despite the large number of dead cells included in the samples (Fig. 4). In contrast, the Ct values of the untreated mixtures increased only slightly with decreasing numbers of viable cells and remained nearly unchanged in mixtures with fewer viable cells (Fig. 4). These results demonstrate that the Ct values of the PMA-treated mixtures were solely dependent on the amount of DNA from viable cells; thus, the PMA treatment effectively inhibited amplification of DNA from dead cells.

### Application of PMA/real-time PCR to artificially infected seeds

To determine whether the PMA/real-time PCR assay could be successfully applied to detect viable *A. citrulli* in infected seeds, healthy watermelon seeds inoculated with different concentrations of viable cells (8 × 10^6^ to 8 × 10 CFU/mL), and 8 × 10^6 ^CFU/mL dead cells were used. Three sets of seed samples (300 seeds per set) from each treatment group were tested using real-time PCR and PMA/real-time PCR, respectively. The results were consistent with those achieved for bacterial cultures. The Ct values of the PMA-treated seeds increased as the actual number of viable cells increased (Table 1). Conversely, the Ct values of the untreated seeds remained nearly unchanged in the case of fewer viable cells (Table 1). These results demonstrated that PMA inhibited the amplification of dead bacteria and that the PMA/real-time PCR assay selectively detected viable *A. citrulli* in infected seeds, without interference from the seed matrix.

### Application of PMA/real-time PCR to naturally infected seeds

To determine whether the PMA/real-time PCR assay could be successfully applied to actual samples from the field, three naturally infected watermelon seed samples that had been treated with 1% HCl (for sterilization) or left untreated were used. To ensure that the naturally infected seed samples carried viable *A. citrulli*, we first tested the seed samples using the seedling grow-out method, which defined the incidence of BFB as approximately 16%, 23%, and 29%, respectively. Both HCl treated and untreated samples were then confirmed to either contain or be free of viable *A. citrulli* by isolation on semi-selective media. The results showed that after approximately 5 days, *A. citrulli* could be successfully isolated and identified from all three HCl untreated samples on semi-selective media, but not from the three HCl treated samples, indicating that HCl effectively kills pathogens on watermelon seeds. For the three naturally infected watermelon seed samples, similar Ct values were observed for HCl untreated samples by real-time PCR and PMA/real-time PCR, and for HCl treated samples by real-time PCR (Table 2). In contrast, the Ct value of the three HCl treated samples obtained by PMA/real-time PCR were all higher than 37 (Table 2). No reactions were observed from both of the two negative controls. In our practical testing work, we usually consider that the results are negative when the Ct values are higher than 35. These results indicate that the PMA/real-time PCR assay is able to successfully and selectively detect viable *A. citrulli* in naturally infected seeds, while avoiding the overestimation of bacterial cells that occurs when using classical PCR. Thus, this PMA/real-time PCR assay is suitable for the detection of viable *A. citrulli* in seed samples from the field.

## Discussion

Since the first report of watermelon bacterial fruit blotch caused by *A. citrulli* in 1990 in China, this disease has been reported in Sinkiang, Ningxia, Inner Mongolia, Hebei, Henan, Shandong, Jiangsu, Nanjing, Guangdong, Fujian, Hainan, Jilin, and Beijing. Economic losses owing to watermelon bacterial fruit blotch result from seed yield reductions and loss of fruit marketability. Effective control of the spread of this disease relies on the availability of sensitive and specific methods that can be used for rapid and accurate detection of the pathogen. Although several methods are available for the detection of *A. citrulli*, these methods are still accompanied by limitations. Therefore, we developed a method for the selective detection of viable, seed-borne *A. citrulli* using a combination real-time fluorescent PCR and PMA treatment. Our work demonstrated that this assay could specifically and sensitively detect viable *A. citrulli* in both pure cultures and infected seeds.

One of the key aspects of our study was the development of an assay using a unique *A. citrulli* sequence as the sole genetic marker for the target in real-time PCR. Several PCR primers have been designed for *A. citrulli* based on 16S ribosomal DNA (rDNA), the 16S-23S internally transcribed spacer (ITS) regions of rDNA, the BOX fragment, and *hrp* gene sequences^11^. Currently, the most commonly used primer set for the detection of *A. citrulli* is WFB1/WFB2 (based on 16S rDNA), which is highly sensitive but also amplifies non-target bacterial DNA including that of *A. avenae*, *A. konjaci*, *A. cattleyae*, *Comamonas testeroni*, and an *Acidovorax* sp. from *Calathea* spp.^20^. Primers SEQID4 and SEQID5 were designed from the 16S-23S ITS region of *A. citrulli*^21^. SEQID4 was subsequently modified by removing the nucleotides “TC” from the 5′ end to yield SEQID4m^22^. Both the original and modified primers are highly specific for *A. citrulli* but are also susceptible to false positive results. In comparison, primers BX-L1 and BX-S-R2 were designed from a BOX-PCR fragment of *A. citrulli* and were reported to be highly specific to *A. citrulli* without compromising detection sensitivity under high-stringency conditions^23^. The TaqMan real-time PCR assay described in this study offers a remedy for the limitations of the PCR assays mentioned above, as demonstrated by several lines of evidence from the specificity tests. First, the current assay was considered highly specific because it did not amplify template DNA from 23 non target bacterial strains including the closely related *Acidovorax* species. In addition, the primers allowed detection of all 116 strains of *A. citrulli* tested, which originated from various locations and from different hosts. Furthermore, the real-time PCR method exhibits several advantages over classical PCR such as providing results in real-time and without the requirement for agarose gel electrophoresis.

Another focus of our study involved differentiating viable *A. citrulli* cells from dead cells. Several PCR-based methods have been reported for detection of *A. citrulli*; however, these assays detect total DNA derived from both viable and dead cells. In recent years, PMA and ethidium monoazide (EMA) have been used in PCR to overcome this problem. A combination of EMA and PCR (using primer set WFB1/WFB2) was developed for the selective detection of *A. citrulli* by Feng *et al.*^24^. Both EMA and PMA are able to intercalate double-stranded DNA or RNA and then irreversibly cross-link to the nucleic acids following photoactivation^12^,^25^. Some studies have observed that EMA, but not PMA, was able to penetrate into viable cells and was toxic to viable cells^12^,^13^. However, the PCR primer sets used in these studies are unable to distinguish *A. citrulli* from the other closely related *Acidovorax* species.

In the current study, we have assessed the capability of PMA/real-time PCR for the differentiation of viable *A. citrulli* from dead cells. Comparison of samples between PMA-treated or non-treated viable or dead *A. citrulli* cells, or a mixture of viable/dead cells, showed almost no difference between the PMA-treated viable cells and non-treated cells, illustrating that the amplification of DNA from viable cells was not affected by PMA treatment. We also demonstrated that the PMA/real-time PCR assay could efficiently prevent DNA amplification from dead cells. Furthermore, we were able to selectively detect viable cells from mixtures with a large number of dead cells (8 × 10^6 ^CFU/mL) using this assay. These results confirmed that the PMA/real-time PCR assay provides a reliable means to determine the presence of viable cells of *A. citrulli*.

We further applied this PMA/real-time PCR assay for the selective detection of viable *A. citrulli* in seeds. Because the bacterium is spread by infected seeds, the only method to effectively control the disease is the use of healthy seeds^26^. Therefore, we applied this assay to selectively detect viable cells of *A. citrulli* in infected seeds. Our results demonstrated that this assay could positively detect viable cells of *A. citrulli* from both artificially and naturally infected seeds. However, during the detection process of the sterilized infected seeds using PMA treatment, fluorescence signals were also collected after 37 cycles when there was a high *A. citrulli* carrier rate. Therefore, we speculate that the amplification of DNA from dead cells was not completely blocked by PMA treatment. A similar phenomenon was also observed by Elizaquivel *et al.*^17^. However, according to our usual testing standards, the samples would be judged as negative for Ct values above 35. Therefore, dead cells of *A. citrulli* on sterilized infected seeds would not be detected as positive findings using the PMA/real-time PCR assay, thus avoiding the overestimation of bacterial cells that occurs when using standard PCR-based methods. As a result, our method should prevent the overestimation of potential disease risk. In addition, we also detected natural infected seeds and those that had undergone sterilization using the traditional agar plating method. The results from this assessment were consistent with those of PMA/real-time PCR, indicating that viable cells of *A. citrulli* could be isolated from naturally infected seeds, whereas no *A. citrulli* strains were isolated from the sterilized infected seeds. However, this isolation method required more than 5 days. In contrast, the PMA/real-time PCR assay was simpler and more rapid, requiring only 1 day. Thus, the PMA/real-time PCR assay possesses marked advantages for the detection of viable cells of *A. citrulli* in seed samples.

In summary, we have developed a new PMA/real-time PCR assay that has been proven to be sensitive and specific for the selective detection of viable cells of *A. citrulli* from infected seeds. Thus, this method could likely be useful for pathogen detection in seeds or to evaluate seed sterilization.

## Materials and Methods

### Bacterial strains and culture conditions

We utilized 116 *A. citrulli* strains and 23 additional strains in our study; the relevant information and sources are listed in Supplementary Table S1. All strains were routinely grown in nutrient broth (NB) for 24–48 h at 28 °C. To enumerate bacterial cells, cultures were serially diluted in 10-fold increments with medium, plated on nutrient agar (NA) plates, and incubated overnight at 28 °C.

### Primer design and real-time PCR conditions

To develop stable and specific primers and a probe for the identification of *A. citrulli*, we evaluated several *A. citrulli* genes (AAC00-1, NC_008752.1) by BLAST analysis to identify an *A. citrulli*-specific sequence. A unique sequence (Aave_1909) encoding a hypothetical protein was identified as a specific genetic marker for *A. citrulli*. Based on this sequence, forward and reverse primers (*A. citrulli*-FP and *A. citrulli*-RP) and also a TaqMan probe (*A. citrulli*-P) were designed using Primer Express 3.0 (Applied Biosystems, Foster City, CA, USA). The expected product size was 121 bp.

*A. citrulli*-P was labelled at the 5′ and 3′ ends with 6-caboxyfluorescein (FAM) and black hole quencher (BHQ1), respectively. The sequences of the primers and probe were as follows: *A. citrulli*-FP: 5′-CTGATAATCCTCGGCTCAACAA-3′; *A. citrulli*-RP: 5′-TGAGCGCATTTCTGACGAG-3′; *A. citrulli*-P: FAM-AAGAAATACGCCCTCGCCAATCTCC-BHQ1. Oligonucleotides were synthesized by Shanghai Huirui Biotechnology (Shanghai, China).

PCR mixtures contained 10 μL 2× qPCR Master Mix (Shanghai Huirui Biotechnology), 0.8 μL each primer (10 μM), 0.4 μL probe (10 μM), and 1 μL template DNA or pure bacterial culture suspension, and an appropriate volume of ddH_2_O was added to reach a reaction volume of 20 μL. Amplifications were performed on the ABI Prism 7900 HT Sequence Detection System (Applied Biosystems, Foster City, CA, USA) using the following thermal cycling parameters: 95 °C for 10 min, and 40 cycles at 95 °C for 15 s and 58 °C for 1 min. Fluorescence was measured at the end of each extension step. Threshold cycles were generated automatically by ABI 7900 HT Prism software. All experiments included a non-template control in which 1 μL ddH_2_O was used rather than the DNA template. Each experiment was performed in triplicate.

### Specificity and sensitivity assays

To test specificity of the real-time PCR reaction, 116 strains of *A. citrulli* from different origins and hosts, 7 closely related *Acidovorax* strains, and 16 strains from another 9 genera were used (Supplementary Table S1). All strains were grown in NB for 24 to 48 h and 1 μL of culture suspension was used directly for real-time PCR as described above.

To determine the real-time PCR assay sensitivity, the culture of *A. citrulli* strain tw24 was determined to be equivalent to 8 × 10^8 ^CFU/mL by plating and was then diluted in sterile water at 10-fold increments to generate serial dilutions ranging from 8 × 10^8^ to 8 × 10 CFU/mL. Each dilution (1 μL) was used for real-time PCR to generate the standard curve for the detection limit.

### Preparation of viable and dead cell mixtures for PMA/real-time PCR

The culture of *A. citrulli* strain tw24 was grown in NB at 28 °C with shaking at 180 rpm to mid-exponential phase and then adjusted to approximately 8 × 10^6 ^CFU/mL by plating. The bacterial suspension was divided into two aliquots. One aliquot was boiled in a water bath for 10 min to kill the cells and then 100 μL of the boiled suspension was spread on an NA plate and incubated at 28 °C for 24–48 h to check cell viability. The other aliquot was used to generate two series of 10-fold dilutions ranging from 8 × 10^6^ to 8 × 10 CFU/mL. One set was used to generate the various samples containing different number of viable cells (8 × 10^6^ to 8 × 10 CFU/mL). The other set was combined with 8 × 10^6 ^CFU/mL dead cells to generate the cell mixtures, which consisted of different numbers of viable cells (8 × 10^6^ to 8 × 10 CFU/mL) along with 8 × 10^6 ^CFU/mL dead cells. Each sample, containing dead cells, different numbers of viable cells, or cell mixtures, was divided into two aliquots and processed as PMA-treated and untreated cell populations.

### PMA treatment and DNA isolation

PMA (Biotium Inc., Hayward, CA, USA) was dissolved in dimethyl sulfoxide to obtain a stock solution of 1 mg/mL and was stored at −20 °C in the dark. PMA (1.5 μL) was added to 500 μL of the viable, heat-killed, and mixed samples described above to final concentrations of 3 μg/mL^14^,^27^. The PMA-treated samples were incubated at room temperature in the dark for 5 min with occasional mixing. Subsequently, the samples were exposed to a 650 W halogen light for 5 min to cross-link the intercalated PMA with DNA. DNA was then isolated by boiling in a water bath for 10 min, followed by rapid cooling on ice for 5–10 min. The samples were centrifuged at 8,000 × *g* for 2–3 min and the DNA-containing supernatant was transferred to a new centrifuge tube for real-time PCR.

### Artificially and naturally infected seed assay

Watermelon seeds artificially or naturally infected with *A. citrulli* were used to evaluate the efficiency of PMA/real-time PCR to distinguish between viable and dead cells in seed samples. The healthy watermelon seeds (Daguoxinxiu, Beijing) used in this assay were first confirmed to be free of *A. citrulli* by traditional agar plating methods. For artificial inoculation, six clean watermelon seed samples were incubated in a set of mixtures containing different concentrations of viable *A. citrulli* cells (8 × 10^6^ to 8 × 10 CFU/mL) as well as 8 × 10^6 ^CFU/mL dead cells for 1 h, respectively, and then were air-dried. Next, 3 sets of seed samples (300 seeds per set) from each treatment group (watermelon seeds infected with 8 × 10^6^ to 8 × 10 CFU/mL viable cells along with 8 × 10^6 ^CFU/mL dead cells) were then tested using real-time PCR and PMA/real-time PCR, respectively.

For the naturally infected seed assay, three infected watermelon seed samples (Daguoxinxing, Hebei; Daguojiaomei, Hebei; Ribenjiaomei, Hebei) were used. All the naturally infected samples were first tested by a seedling grow-out method to determine the incidence of BFB. These were then split into two equivalent samples; one remained untreated, and the other was treated with 1% HCl to kill the pathogen. The infected seeds were soaked in 1% HCl for 10 min and then rinsed with water 4–5 times until the pH value reached 7. Both HCl treated and untreated naturally infected seed samples were tested by traditional agar plating methods to be confirmed as free or containing viable *A. citrulli*. Next, 300 seeds per treatment group (HCl treated and untreated) of the three naturally infected seed samples were tested using real-time PCR and PMA/real-time PCR, respectively. Tow healthy seed samples that treated with only 1% HCl or treated with only ddH_2_O were also tested as the negative control.

For traditional isolation, the seeds were soaked in seed extract buffer at 4 °C overnight and then 100 μL of undiluted and 10^−1^ dilutions of seed wash were spread onto selective media (EBBA) and incubated at 37 °C for 5 days, as described by Zhao *et al.*^28^. For real-time PCR and PMA/real-time PCR assays, each set of seed samples (300 seeds per set) was incubated in sterile distilled water for 4 h. Then, 3 mL seed extract was centrifuged at 10,000 × *g* for 5 min and the pellets were resuspended in 1 mL ddH_2_O. The suspensions of each sample were split into two equivalent samples and 500 μL was treated with PMA as described above and 500 μL was left untreated. Both PMA-treated and non-treated suspensions were centrifuged at 10,000 × *g* for 2 min. Pellets were used for DNA extraction using the DNA secure Plant Kit (Tiangen Biotech, Beijing, China) according to the manufacturer’s instructions. Finally, each DNA sample (1 μL) was used for real-time PCR. 1 μL water was uesd to substitute template DNA to serve as non-template control.

## Additional Information

**How to cite this article**: Tian, Q. *et al.* Selective detection of viable seed-borne *Acidovorax citrulli* by real-time PCR with propidium monoazide. *Sci. Rep.* **6**, 35457; doi: 10.1038/srep35457 (2016).

## Supplementary Material

Supplementary Information

## Figures and Tables

**Table 1:** Effect of propidium monoazide (PMA) treatment on cycle threshold (Ct) values obtained in real-time PCR assays for artificially infected seeds.

Artificially infected seeds viable cells/dead cells (CFU/mL)	Ct value	
	Not treated with PMA	PMA treated
8 × 10^6^/8 × 10^6^	20.25^a^	21.89
8 × 10^5^/8 × 10^6^	24.44	25.22
8 × 10^4^/8 × 10^6^	27.47	29.34
8 × 10^3^/8 × 10^6^	29.37	32.57
8 × 10^2^/8 × 10^6^	29.45	36.82
8 × 10/8 × 10^6^	29.97	40

^a^Numbers represent the means of Ct values of three replications for each treatment.

**Table 2:** Effect of propidium monoazide (PMA) treatment on cycle threshold (Ct) values obtained in real-time PCR assays for naturally infected seeds.

Naturally infected seeds	Ct value	
	Not treated with PMA	PMA treated
Sample 1 (untreated)	27.18	28.28
Sample 1 (treated with 1% HCl)	29.31	37.74
Sample 2 (untreated)	28.99	29.51
Sample 2 (treated with 1% HCl)	30.19	38
Sample 3 (untreated)	29.16	29.89
Sample 3 (treated with 1% HCl)	31.33	38
