# Supplementary material for: Selective detection of viable seed-borne Acidovorax citrulli by real-time PCR with propidium monoazide
Source: Sci Rep. 2016 Oct 14;6:35457. doi: 10.1038/srep35457 (PMC5064318; doi:10.1038/srep35457)
Supplement: Supplementary Information [file srep35457-s1.pdf]

Selective detection of viable seed-borne *Acidovorax citrulli* by real-time PCR with propidium monoazide

Qian Tian[1], Jian-jun Feng[2,4], Jie Hu[3], Wen-jun Zhao[1*]

[1]Chinese Academy of Inspection and Quarantine, Beijing 100176, China

[2]Shenzhen Entry-Exit Inspection and Quarantine Bureau, Shenzhen 518045, China

[3]Shaanxi University of Technology, Shaanxi 723001,China

[4]Shenzhen Academy of Inspection and Quarantine, Shenzhen 518010,China

*Corresponding author: Wen-jun Zhao

Chinese Academy of Inspection and Quarantine, Yizhuang Economic and Technological Development Zone, Daxing District, No. 11 Ronghua South Road, Beijing 100176, China

Phone: +86-10-53897557

E-mail: wenjunzhao@188.com

Supplementary Table S1. Bacterial strains used in this study.

No.	Species	Strain	Origin	Host	Source	Real-time PCR results
1	*Acidovoraxcitrulli*	ATCC29625	USA	watermelon	ATCC	+
2		00-1	USA/Georgia	watermelon	Baishi Hu	+
3		94-36	USA/Georgia	watermelon	Baishi Hu	+
4		94-21	USA/Georgia	watermelon	Baishi Hu	+
5		92-17	USA/Florida	watermelon	Baishi Hu	+
6		94-55	USA/Georgia	watermelon	Baishi Hu	+
7		94-87	USA/Georgia	watermelon	Baishi Hu	+
8		94-39	USA/Georgia	watermelon	Baishi Hu	+
9		Saticoy B Hopking	USA/Florida	watermelon	Baishi Hu	+
10		94-48	USA/Georgia	watermelon	Baishi Hu	+
11		201-12	USA/South Carolina	watermelon	Baishi Hu	+
12		94-12	USA/Georgia	watermelon	Baishi Hu	+
13		203-50	Chile	cantaloupe	Baishi Hu	+
14		206-102	China	watermelon	Baishi Hu	+
15		206-103	China	watermelon	Baishi Hu	+
16		208-27	USA/Indiana	watermelon	Baishi Hu	+
17		205-14	USA/Indiana	watermelon	Baishi Hu	+
18		92-301	USA/Georgia	watermelon	Baishi Hu	+
19		92-305	USA/Georgia	watermelon	Baishi Hu	+
20		92-300	USA/Georgia	watermelon	Baishi Hu	+
21		201-18	Australia	watermelon	Baishi Hu	+
22		202-1	China	muskmelon	Baishi Hu	+
23		98-17	USA/Georgia	pumpkin	Baishi Hu	+
24		99-5	USA/Georgia	cantaloupe	Baishi Hu	+
25		200-23	USA/Oklahoma	watermelon	Baishi Hu	+
26		200-6	USA/Georgia	watermelon	Baishi Hu	+
27		200-30	USA	cantaloupe	Baishi Hu	+
28		201-13	Australia	watermelon	Baishi Hu	+
29		202-66	Israel	melon	Baishi Hu	+
30		203-55	Honduras	watermelon	Baishi Hu	+
31		207-41	China	watermelon	Baishi Hu	+
32		206-2	China	watermelon	Baishi Hu	+
33		206-1	China	watermelon	Baishi Hu	+
34		206-75	China	watermelon	Baishi Hu	+
35		206-95	China	watermelon	Baishi Hu	+
36		206-79	China	watermelon	Baishi Hu	+
37		208-7	China	watermelon	Baishi Hu	+

38		Xj121	China/Sinkiang	watermelon	Baishi Hu	+
39		Njl12	China/Sinkiang	watermelon	Baishi Hu	+
40		NXWF06	China/Ningxia	watermelon	Baishi Hu	+
41		NM01	China/Inner Mongolia	watermelon	Baishi Hu	+
42		XjL06	China/Sinkiang	watermelon	Baishi Hu	+
43		Xjl12	China/Sinkiang	watermelon	Baishi Hu	+
44		HB01	China/Hebei	watermelon	Baishi Hu	+
45		Bt101	China/Sinkiang	watermelon	Baishi Hu	+
46		SD01	China/Shandong	watermelon	Baishi Hu	+
47		SD02	China/Shandong	watermelon	Baishi Hu	+
48		Fc440	China	watermelon	Baishi Hu	+
49		NX01	China/Ningxia	watermelon	Jianping Yi	+
52		SY13	China	watermelon	Jianping Yi	+
53		Boo1	China/Jiangsu	watermelon	Jianping Yi	+
54		Aac98-10	China	watermelon	Jianping Yi	+
55		tw24	China	watermelon	Tingchang Zhao	+
56		tw31	China	watermelon	Tingchang Zhao	+
57		tw33	China	watermelon	Tingchang Zhao	+
58		pslb19	China	muskmelon	Tingchang Zhao	+
59		pslb91	China	muskmelon	Tingchang Zhao	+
60		pslb102	China	muskmelon	Tingchang Zhao	+
61		LS-2	China/Hainan	watermelon	LifangZou	+
62		LS-3	China/Hainan	watermelon	LifangZou	+
63		LS-5	China/Hainan	watermelon	LifangZou	+
64		XJ-1	China/Sinkiang	watermelon	LifangZou	+
65		XJ-3	China/Sinkiang	watermelon	LifangZou	+
66		XJ-4	China/Sinkiang	watermelon	LifangZou	+
67		XJ-5	China/Sinkiang	watermelon	LifangZou	+
68		XJ-6	China/Sinkiang	watermelon	LifangZou	+
69		Sy-1	China	watermelon	LifangZou	+
70		Sy-3	China	watermelon	LifangZou	+
71		Sy-4	China	watermelon	LifangZou	+
72		ZZ-1	China	watermelon	LifangZou	+
73		PSLB25	China	muskmelon	LifangZou	+
74		PSLB29	China	muskmelon	LifangZou	+
75		PSLB-96	China	muskmelon	LifangZou	+
76		PSLBtw20	China	muskmelon	LifangZou	+
77		FC455	China	watermelon	LifangZou	+
78		Aac21	China/Guangdong	watermelon	LifangZou	+
79		PSLB1	China/Inner Mongolia	muskmelon	LifangZou	+
80		PSLB10	China/Inner	muskmelon	LifangZou	+

			Mongolia			
81		PSLB37	China/Sinkiang	muskmelon	LifangZou	+
82		Xj104	China/Sinkiang	watermelon	LifangZou	+
83		Xj13	China/Sinkiang	watermelon	LifangZou	+
84		Xj101	China/Sinkiang	watermelon	LifangZou	+
85		HN01	China/Hainan	watermelon	LifangZou	+
86		B	USA	watermelon	LifangZou	+
87		C	USA	watermelon	LifangZou	+
88		D	USA	watermelon	LifangZou	+
89		E	USA	watermelon	LifangZou	+
90		F	USA	watermelon	LifangZou	+
91		G	USA	watermelon	LifangZou	+
92		H	USA	watermelon	LifangZou	+
93		I	USA	watermelon	LifangZou	+
94		J	USA	watermelon	LifangZou	+
95		K	USA	watermelon	LifangZou	+
96		L	USA	watermelon	LifangZou	+
97		M	USA	watermelon	LifangZou	+
98		N	USA	watermelon	LifangZou	+
99		SM1	USA	watermelon	LifangZou	+
100		SW1	USA	watermelon	LifangZou	+
101		SMb	USA	watermelon	LifangZou	+
102		SWb	USA	watermelon	LifangZou	+
103		A8	China/Nanjing	watermelon	Baishi Hu	+
104		Btc-29	China/Nanjing	watermelon	Baishi Hu	+
105		B8	China/Nanjing	watermelon	Baishi Hu	+
106		BJ-A	China/Beijing	watermelon	LifangZou	+
107		BJ-B	China/Beijing	watermelon	LifangZou	+
108		BJ-C	China/Beijing	watermelon	LifangZou	+
109		DE1	China	watermelon	Tingchang Zhao	+
110		FC183	USA	watermelon	Norm.W.Scchad	+
111		FC248	USA	watermelon	Norm.W.Scchad	+
112		FC374	USA	watermelon	Norm.W.Scchad	+
113		FC526	Australia	watermelon	LifangZou	+
114		LS-10	China/Hainan	watermelon	LifangZou	+
115		ZX3L-03	China	watermelon	Baishi Hu	+
116		7716	China	watermelon	LifangZou	+
117	*Acidovorax avenae* subsp. *avenae*	ATCC19307	Reunion	sugarcane	ATCC	-
118	*Acidovoraxcattleyae*	ATCC 10200	USA	cattleya	ATCC	-
119		NCPPB 961	unknown	unknown	NCPPB	-
120		NCPPB 4196	Brazil	*Phalaenopsis* hybrid	NCPPB	-

121		NCPPB 4198	Brazil	*Orchidaceae*	NCPPB	-
122		NCPPB 4200	Brazil	*Orchidaceae*	NCPPB	-
123	*Acidovorax konjaci*	ATCC 33996	Japan	konjak	ATCC	-
124	*Burkholderia andropogonis*	ATCC 23060	USA	corn	ATCC	-
125	*Burkholderia gladioli*	ATCC 10248	unknown	*Gladiolus sp.*	ATCC	-
126	*Clavibacter michiganense* subsp. *michiganense*	ATCC 14456	Italy	tomato	ATCC	-
127	*Curtobacterium flaccum faciens*	ATCC 7392	unknown	unknown	ATCC	-
128	*Erwinia billingiae*	DSMZ 17872	unknown	unknown	DSMZ	-
129	*Erwinia tracheiphila*	NCPPB2133	unknown	*Cucumis sativus*	ATCC	-
130	*Pantoea agglomerans*	ATCC13329	unknown	unknown	ATCC	-
131	*Pectobacterium carotovorum* subsp. *carotovorum*	ATCC138	unknown	unknown	ATCC	-
132	*Pseudomonas syringae* pv. *lachrymans*	ATCC7386	USA	cucumber	ATCC	-
133		NCPPB540	Denmark	*Cucumis sativus*	NCPPB	-
134		NCPPB467	UK	*Cucumis sativus*	NCPPB	-
135		NCPPB1425	Hungary	*Cucumis sativus*	NCPPB	-
136		NCPPB1096	Hungary	*Cucumis sativus*	NCPPB	-
137	*Rhizobium radiobacter*	ATCC19358	unknown	unknown	ATCC	-
138	*Xanthomonas axonopodis* pv. *citri*	X206	China/Taiwan	citrus	Jianping Yi	-
139	*Xanthomonas cucurbitae*	NCPPB2597	New Zealand	*Cucurbita maxima*	NCPPB	-

ATCC, American Type Culture Collection, Manassas, VA, USA

NCPPB, National Collection of Plant Pathogenic Bacteria

DSMZ, Deutsche Sammlung von Mikroorganismen und Zellkulturen

+, positive results in the real-time PCR assay

-, failed to react in the real-time PCR assay
